# Supplementary material for: Causal Relationship between Aging and Anorexia Nervosa: A White-Matter-Microstructure-Mediated Mendelian Randomization Analysis
Source: Biomedicines. 2024 Aug 16;12(8):1874. doi: 10.3390/biomedicines12081874 (PMC11351342; doi:10.3390/biomedicines12081874)
Supplement: Supplementary file 1 [file biomedicines-12-01874-s001.zip › Supplementary Figure/Figure S7/Single SNP plot of MR analysis for ubm-b-1942 to AN.pdf]

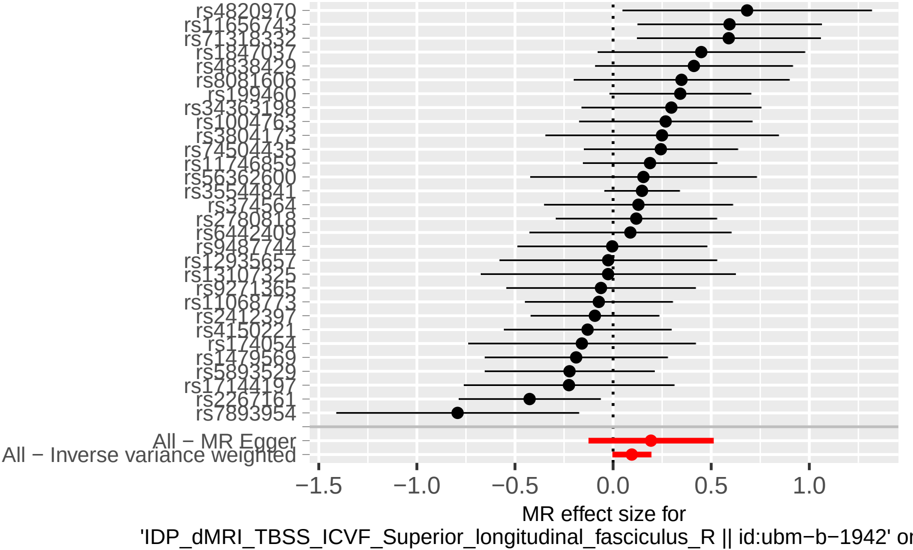
rs4820970
rs11656743
rs71318332
rs1847037
rs4838429
rs8081606
rs199460
rs34363198
rs1004763
rs3804173
rs74504435
rs11746859
rs56362600
rs35544841
rs374564
rs2780818
rs6442409
rs9487744
rs12935657
rs13107325
rs9271365
rs11068773
rs2412397
rs4150221
rs174054
rs1479569
rs5893529
rs17144197
rs2267161
rs7893954
All − MR Egger
All − Inverse variance weighted
−1.5
−1.0
−0.5
0.0
0.5
1.0
MR effect size for
'IDP_dMRI_TBSS_ICVF_Superior_longitudinal_fasciculus_R || id:ubm−b−1942' on
